# Frailty Status, Not Just Age, is Associated With Postoperative Opioid Consumption: A Retrospective, Population-based Analysis

**DOI:** 10.1097/AS9.0000000000000496

**Published:** 2024-10-04

**Authors:** Kyle R. Latack, Ryan Howard, Mark C. Bicket, Samantha Cooley, Vidhya Gunaseelan, Michael Englesbe, Jennifer Waljee

**Affiliations:** From the * Department of Obstetrics and Gynecology, University of Michigan, Ann Arbor, MI; † Department of Surgery, University of Michigan, Ann Arbor, MI; ‡ Michigan Opioid Prescribing Engagement Network, Institute for Healthcare Policy and Innovation, University of Michigan, Ann Arbor, MI; § Center for Health Outcomes and Policy, Michigan Medicine, Ann Arbor, MI; ∥ Department of Anesthesia, University of Michigan, Ann Arbor, MI; ¶ School of Medicine, Kansas City University, Kansas City, MO

**Keywords:** frailty, opioids, surgical patients

## Abstract

**Objective:**

To assess the relationship between postoperative opioid consumption and frailty status.

**Background:**

Physiologic reserve can be assessed through both chronologic age as well as measures of frailty. Although prior studies suggest that older individuals may require less opioid following surgery, chronologic age, and frailty do not always align, and little is known regarding postoperative opioid consumption patterns by frailty.

**Methods:**

We conducted a retrospective analysis of opioid-naïve adult patients undergoing common general, vascular, and gynecologic procedures across a statewide quality improvement program from November 6, 2017 to February 28, 2021. Our primary outcome was postoperative patient-reported opioid consumption within 30 days of surgery in oral morphine equivalents (OME). Our primary exposure was frailty status defined by the modified frailty index (mFi-5) criteria. Other covariates included patient demographic and clinical attributes, procedural factors, discharge opioid prescription size, and postoperative complications. Linear regression was performed to assess the association of frailty status and opioid consumption, stratified by age.

**Results:**

In this cohort of 34,854 patients, 10,596 had an mFi-5 score of ≤1 and 3,635 had a score of >1. A score of >1 was associated with increased patient-reported opioid consumption (OMEs 3.3 greater; 95% CI = 1.5–5.1). This held true for individuals over 65 (OMEs 2.7 greater; 95% CI = 0.2–5.1). Frailty status, regardless of score, was negatively associated with an opioid prescription at discharge.

**Conclusions:**

Frailty status is associated with increased opioid consumption after common operations. Future prescribing guidelines and outcomes analyses should consider this marker when reviewing opioid consumption data and related adverse outcomes.

## INTRODUCTION

Frailty is associated with increased adverse postoperative outcomes, including increased mortality, length of hospital stay, readmission rates, and postoperative complications.^1–3^ Simply defined, frailty is a multidimensional process characterized by decreased physiologic reserve and resistance to stressors and has emerged as a distinct risk factor in addition to chronological aging.^4^ While the association of frailty and clinical outcomes following surgical procedures has been described, much less is known regarding the potential interplay of frailty on postoperative pain management.

Prior studies have examined opioid prescribing following common surgical procedures, indicating that comorbid conditions and age are associated with both variation in prescribing and prolonged postoperative use.^5–8^ Current guidelines acknowledge older individuals as a heterogenous group with considerations for frailty status but how opioid prescriptions should be modified for this population is unclear.^9^ Frailty has also been shown to be a risk factor for adverse drug reactions in general.^10,11^ More broadly, frailty has been shown to be an independent risk factor for intrusive pain (pain that interferes with daily activity),^12^ and amplified by the presence of chronic medical conditions.^13^ Therefore, adequate, evidence-based, pain control after surgery is critical as pain could be exacerbated perioperatively. Despite this, few studies have examined frailty as a unique risk factor for pain, and there is limited data on frail individuals’ postoperative opioid needs.

Our primary outcome was to assess whether frailty was associated with the amount of postoperative opioids consumed. We hypothesized that frail individuals would consume more opioids even when controlling for age due to the interaction of pain and existing comorbidities. Our secondary outcome was to assess differences in opioid prescribing patterns for frail individuals. Due to comorbidities, providers may be hesitant to prescribe opioids for frail individuals, and therefore we hypothesize frail individuals are less like to receive a postoperative opioid prescription. This understanding can help develop stronger evidence-based prescribing guidelines for frail individuals particularly as the population continues to age in the United States.

## METHODS

The primary data source for patient and procedure data is the Michigan Surgical Quality Collaborative (MSQC). The MSQC is a clinical registry that prospectively collects data on patients for general surgery operations as well as vascular and gynecologic operations across hospitals in Michigan. Data within this registry includes patient demographic information, perioperative care, and 30-day clinical outcomes. In January 2017, the MSQC started to collect data on opioid prescriptions at the time of discharge from surgery as well as patient-reported opioid consumption and refills. Patients were contacted between 30 and 90 days after surgery and asked to report how many opioid pills they consumed and if they had refilled an opioid prescription.^14^

The secondary data source was the prescription drug monitoring program (PDMP) of the state of Michigan. The PDMP contains data on all the controlled substances prescription fills in the state. MSQC data were linked with data from PDMP using a state-approved process by an independent third party. Patient identifiers were removed by the independent third party and replaced by encrypted identifiers before the delivery of data to the study team. The data from the PDMP was used to determine if the patient had filled an opioid prescription in the year before admit to surgery.

All patients enrolled in MSQC ages 18 years and older admitted and having surgery between November 6, 2017 and February 28, 2021 and having data on occurrence and type of discharge opioid prescription were included in the study. We excluded patients with prolonged hospital admission as defined by length of stay greater than 14 days, were not discharged home, died within 30 days of surgery, lived outside Michigan, and had more than 1 match in the PDMP. We also excluded any patient who had filled an opioid prescription 365 days to 31 days before admission for surgery, denoted by the presence of an opioid prescription in the PDMP, so that data was not confounded by recent opioid exposure. Finally, we also excluded patients who did not have valid or complete data for the main explanatory variable or outcomes.

Our primary outcome was patient-reported opioid consumption (in oral morphine equivalents [OME]) among patients who received an opioid prescription at discharge. Secondary outcomes included the following: provision of a discharge opioid prescription, refilling an opioid prescription, and consuming all opioids prescribed.

The main independent variable of interest was frailty status as defined by the Modified Frailty Index-5 (mFi-5) criteria.^15^ This is a previously defined marker for frailty and is a composite score based on 5 clinical criteria: congestive heart failure, diabetes, totally or partially dependent functional status, chronic obstructive pulmonary disease, and hypertension. These clinical criteria were obtained from MSQC data and a composite score was generated. Frailty was stratified by scores of zero, 1, and 2 or more.^16,17^ Additionally, we include the following explanatory variables: age (less than 45, 45–64, and greater than/equal to 65), gender, race/ethnicity, insurance, American Society of Anesthesiologists class, body mass index, cancer, tobacco use, inpatient surgical status, surgical priority, procedure type, size of the discharge opioid prescription, postoperative complications, readmissions, emergency room visits and reoperations at 30 days after surgery. Opioid-related emergency room visits and readmissions were also coded specifically to quantify such outcomes but were not included in regression analysis.

Cohort size (ie denominator) was related to the outcome of interest. For consumption data (primary group of interest), only patients who filled a discharge prescription were included. For discharge prescription, the cohort consisted of all patients undergoing surgery. Finally, refill data was evaluated for all patients who filled a prescription and had valid refill data.

Descriptive statistics were calculated for the study cohort based on 3 categories of frailty scores. χ^2^ or one-way ANOVA tests were used to analyze differences between the group characteristics. To examine the association of frailty on consumption, a multivariable linear regression model with robust standard errors was used controlling for all covariates listed above. We calculated predicted means and 95% confidence intervals (95% CIs) for consumption using this model. A multivariable logistic regression model adjusting for all covariates mentioned above was used to examine the association of frailty and receipt of an opioid prescription at discharge. We calculated the proportion of patients refilling an opioid prescription and the proportion of patients consuming all opioids that they were prescribed. We conducted the above-mentioned analyses, including the sensitivity analysis, in a subset of patients who were 65 years and older. We also performed a sensitivity analysis after excluding patients who did not have an emergency room visit, readmission, complications, and reoperations in the 30 days after surgery. Significance was defined as a *P*-value less than 0.05. All analyses were performed using Stata/SE v17.0 (StataCorp, College Station, Texas).

## RESULTS

**FIGURE 1.**
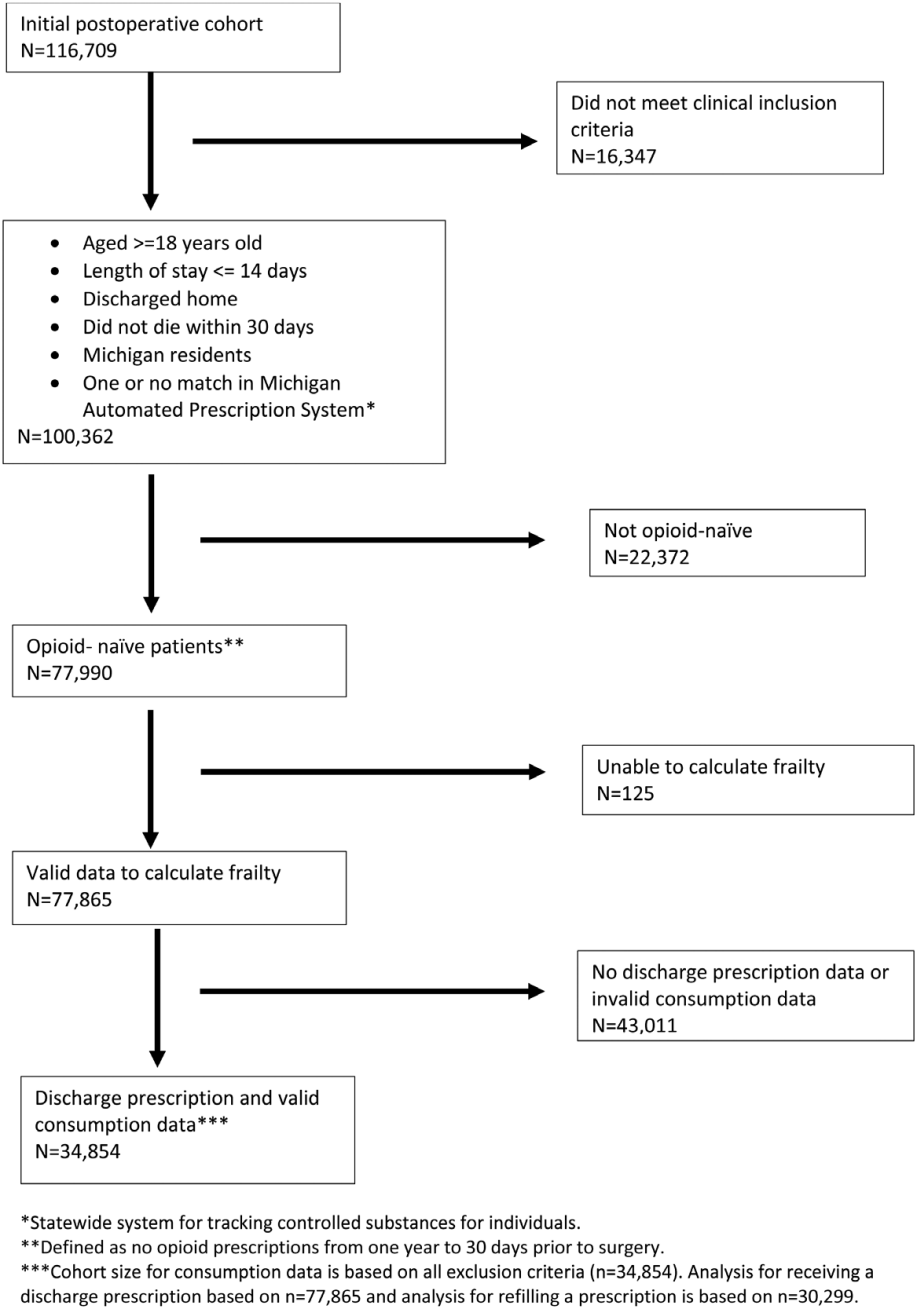
Study cohort flow chart.

A total of 34,854 patients met the inclusion criteria for the study cohort examining opioid consumption by frailty status (Fig. 1). Descriptive statistics of patient characteristics are displayed in Supplement Table 1, http://links.lww.com/AOSO/A407. The average age in our cohort was 52.9 (SD, 16.15) and the majority were individuals identifying as female (55.8%). Within this cohort, 59.2% (n = 20,623) had a frailty score of zero, 30.4% (n = 10,596) had a score of 1, and 10.4% (n = 3635) had a score of greater than or equal to 2. The most common procedures were minor hernia repair (n = 10,314; 29.6%), laparoscopic cholecystectomy (n = 9,312; 26.7%), and laparoscopic appendectomy (n = 3,606; 10.3%). The majority of surgeries were elective (n = 26,575; 76.2%) and performed on an outpatient basis (n = 18,575; 53.3%).

Unadjusted prescription and consumption data are shown in Table 1. The mean opioid prescribed across the cohort was 80.2 OMEs (SD, 60.1). By frailty score, the mean (SD) prescribed opioids were: score zero 78.6 (55.2) score one 81.9 (69.8), and greater than and equal to two 83.8 (62.5). The mean patient-reported consumption across the cohort was 35.2 OMEs (SD, 48.4). By frailty score, the mean (SD) consumed opioids were: score zero 35.4 (45.3), score one 33.7 (50.2), and greater than and equal to two 38.4 (58.9).

**TABLE 1.** Univariate Analysis of Opioid Prescribing and Consumption by Frailty Status

		mFi-5 Criteria Score			
		0	1	≥2	Overall
Received a discharge prescription N(%)	No discharge prescription	6336 (13.8)	4323 (18.6)	1866 (21.9)	12525 (16.1)
	Discharge prescription	39720 (86.2)	18951 (81.4)	6669 (78.1)	65340 (83.9)
	Total	46,056	23,274	8535	77,865
Consumed all opioids prescribed N (%)	No	15031 (72.9)	8075 (76.2)	2635 (72.5)	25741 (73.9)
	Yes	5592 (27.1)	2521 (23.8)	1000 (27.5)	9113 (26.2)
	Total	20,623	10,596	3635	34,854
Refilled a prescription N(%)*	No refill	17335 (95.9)	8755 (96.2)	2961 (95.2)	29051 (95.9)
	Refill	748 (4.1)	349 (3.8)	151 (4.9)	1248 (4.1)
	Total	18,083	9104	3112	30,299
Prescribed OME, mean (SD)		78.63 (55.16)	81.92 (69.81)	83.75 (62.51)	80.16 (60.77)
Mean (SD) consumption [OMEs]		35.4 (45.29)	33.7 (50.19)	38.4 (58.86)	35.2 (48.40)
Median (25–75^th^ percentile) consumption [OMEs]		25 (0–50)	15 (0–50)	20 (0–60)	20 (0–50)

Values for each variable in this table are unadjusted for patient characteristics.

* 30,299 out of 34,854 patients had valid refill data.

**FIGURE 2.**
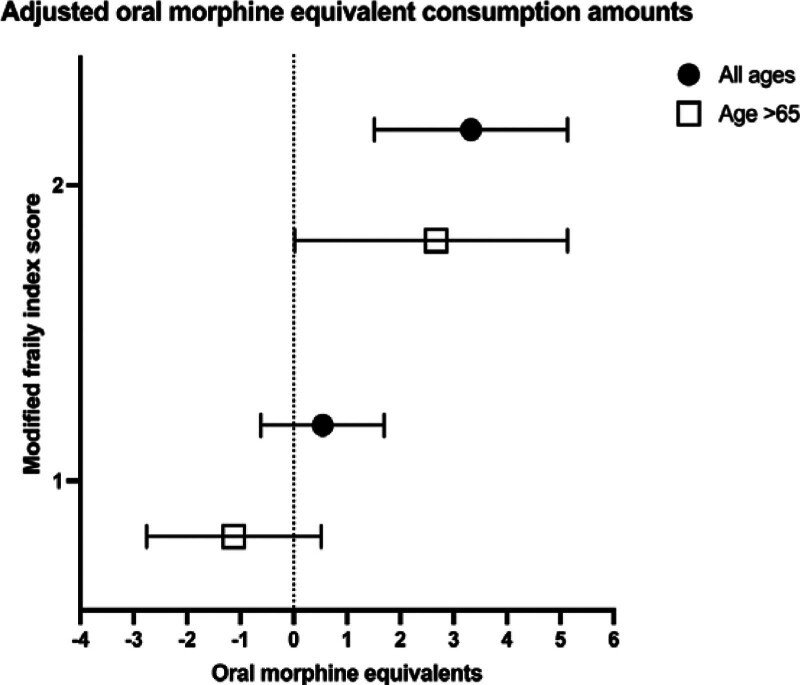
Linear regression results for the adjusted difference in total opioid consumption (in oral morphine equivalents) by frailty group (reference group is mFi-5 score equal to zero). Results shown for all ages and those greater than 65 years old.

In the adjusted analysis, patients with a frailty score of greater than or equal to two for all ages consumed a significantly higher amount of opioids (OME 3.3; 95% CI = 1.5–5.1) (Fig. 2). There was no difference for those with a score of one (OME 0.5; 95%CI = −0.6 to 1.7). Conversely, increasing age was associated with significantly less opioid consumption (>65 years old OME −21.1; 95% CI = −23.3 to −18.8, reference <45 years old). When limiting the analysis to individuals ages 65 and older, we found a similar result where individuals with a frailty score of two or greater consumed a higher amount of opioids (OME 2.7; 95% CI = 0.2–5.1). However, there was no difference for those with a score of one (OME −1.1; 95% CI = −2.8 to 0.5).

**FIGURE 3.**
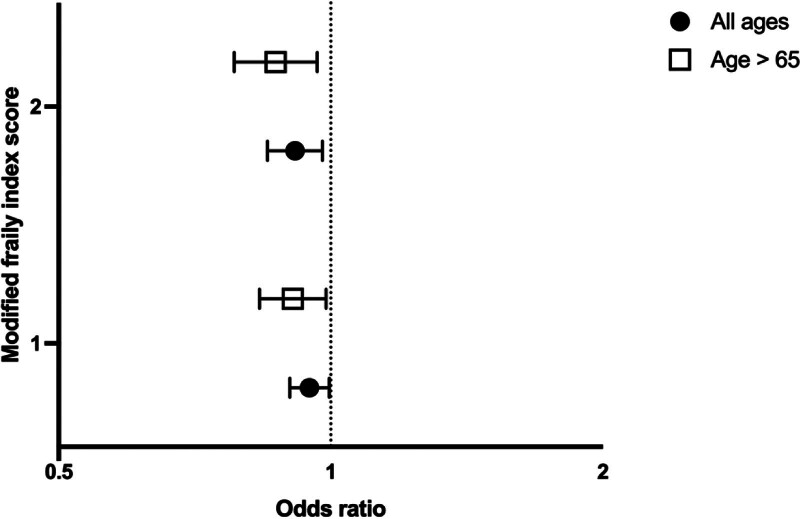
Logistic regression results for adjusted odds of receiving a discharge opioid prescription by frailty group (reference group is mFi-5 score equal to zero). Results shown for all ages and those greater than 65 years old. The X-axis is graphed on log scale.

In our data set, 77,865 were included in our prescription group of which 83.9% (n = 65,340) received a discharge prescription. Across all ages, 78.1% (n = 6669) of those with a frailty score of 2 or greater received a discharge prescription compared with 81.4% (n = 18,951) of those with a score of 1 and 86.2% (n = 39,720) with a score of zero. In the adjusted analysis, patients with a frailty score of either 1 or greater than or equal to 2 had a lower odds of receiving a prescription (OR = 0.95; 95% CI = 0.9–1.0 and OR = 0.91; 95% CI = 0.85–0.98, respectively; Fig. 3). This was also true for individuals who were 65 years or older (OR = 0.91; 95% CI = 0.83–0.99 and OR = 0.87; 95% CI = 0.78–0.97). Additionally, increasing age was associated with significantly lower odds of receiving a discharge prescription (ref <45; 45–64; OR = 0.79; 95% CI = 0.75–0.83;≥65 0.63; 95% CI = 0.58–0.69).

Finally, the majority of patients did not consume all opioids prescribed (n = 25,741;73.9%) and a majority did not refill a prescription (n = 29,051; 95.9%) (Table 1). By frailty score these percentages respectively were: score zero 72.9% and 95.9%, score one 76.2% and 96.2%, and greater than and equal to two 72.5% and 95.2%. We then performed this analysis in those 65 years and older. Within this group, 84.6% (n = 7875) did not consume all opioids prescribed, and 97.5% (n = 7504) did not refill a prescription. By frailty score, 85.8% of those with a score of zero did not consume all prescribed opioids, compared to 85.4% with a score of one and 80.8% of those with two or greater. For refill data, 97.7% of those with a score of zero did not refill a prescription, compared to 97.8% of those with a score of one and 96.5% of those with a score of two or greater.

In the sensitivity analysis excluding patients with complications, readmissions, emergency room visits, and reoperations patients with a frailty score of greater than or equal to two consumed significantly more opioids for all ages (OME = 3.3; 95% CI = 1.5–5.1) and this was true in the analysis of patients 65 years or older (OME = 2.73; 95% CI = 0.24–5.2) (Supplemental Table 2, http://links.lww.com/AOSO/A408 and Supplemental Table 3, http://links.lww.com/AOSO/A409).

## DISCUSSION

In this retrospective analysis of over 30,000 opioid-naive patients undergoing common surgical procedures, individuals with greater frailty scores were less likely to receive an opioid prescription at discharge but consumed slightly higher amounts of opioid in the postoperative period. This finding also holds true in our subset of frail individuals 65 years and older. These results suggest an important opportunity to align prescribing with individual patient needs, particularly for individuals with greater frailty who may be more likely to experience opioid-related complications.

Despite increasing attention to frailty as a risk factor, evidence on postoperative pain management in this population remains limited. To our knowledge, there has only been one prior study examining opioid consumption in frail versus nonfrail individuals in the postoperative period.^18^ This study found that most frail individuals consumed nearly all prescribed opioids, while the majority of nonfrail individuals consumed few to none. Our findings build on this work by utilizing a larger cohort across a wide range of surgical procedures. Aligned with prior work, we again found a difference in consumption patterns. Auckley et al^18^ noted that at 7 days postoperation, frail individuals had higher pain scores. This difference in pain scores could partially be due to the presence of existing intrusive pain or chronic medical comorbidities.^12,13^

The modest consumption difference in our cohort between frail and nonfrail individuals (approximately 3 OMEs) suggests that we may not need to make substantial adjustments for frailty within current treatment guidelines, specifically reduction, but rather monitor closely for adverse events in this population. It is important to note in our cohort, opioid-related emergency room visits and readmissions were both rare and similar between all frailty scores. The unadjusted data suggests a right skew with a smaller group of patients using a higher amount of opioids. This skew is likely at least partially due to certain surgeries requiring higher doses of pain medications postoperatively which was controlled for in our regression. Future studies could expand on these results to assess for variation geographically and in settings with more limited prescribing guidelines as well as identifying additional outlier groups/procedures.

Identification of this difference is important early as undertreated pain can also have an adverse impact on frail individuals. Notably, the presence of chronic pain is related to increased frailty,^19^ and frail individuals with undertreated pain are at increased risk for harm.^13^ Poorly controlled postoperative pain in the acute setting can lead to chronic pain as well as increased morbidity and decreased quality of life and function.^20^ Additionally, at baseline, frail individuals may be at increased risk for chronic pain postoperatively.^21^ All of these risk factors highlight the importance of adequate postoperative pain control for frail individuals. Our data finds that frailty status is an independent risk factor for not receiving an opioid prescription at time of discharge. It is likely a number of those who did not receive an opioid prescription had adequate pain control. However, responsible prescribing patterns, instead of avoidance of prescribing, may offer better outcomes.

The increased risk of adverse events postoperatively for frail individuals across a range of surgeries is well-defined.^1^ Early identification of these individuals is important for risk assessment and decision-making around surgery. This can be done through clinical tasks such as gait speed and Timed-Up-and-Go test, as well as other assessments and questionnaires.^19^ While the mFi-5 score includes “partially or totally dependent functional health status at time of surgery”, other functional status markers were not available, though we would expect similar data trends using other markers of frailty. There appears to be limited data on interventions to improve perioperative outcomes in frail individuals, however.^22,23^ Targeting appropriate pain management may be one avenue for improving outcomes. This would include evidence-based opioid prescribing patterns as well as multimodal pain therapy to limit opioids.

This study is not without limitations. Opioid consumption data was self-reported and therefore susceptible to a recall bias or inaccuracy. However, this is likely adjusted for across a large group of patients, and the true trends are likely similar. Additionally, we did not explore the relationship between opioid consumption and adverse or long-term outcomes. Future studies examining the impact of frailty on adverse opioid-related outcomes are important to understand this relationship. We also were not able to assess if patients had chronic pain preoperatively, however, we attempted to mitigate this effect by excluding persons who were not opioid-naïve before surgery. Finally, we had limited data on patient-reported outcomes within this cohort. Future studies could examine patients’ overall surgical experiences as it relates to frailty and different prescriptions.

## CONCLUSIONS

In this analysis across a wide range of common surgeries, frailty was associated with increased postoperative opioid consumption. Frail individuals are at risk for increased perioperative morbidity and mortality, and opioid consumption may be a modifiable risk factor. Evidence-based prescribing guidelines mindful of this difference are important, and future studies can continue exploring postoperative opioid use in this vulnerable population.

## Supplementary Material
