# Supplementary material for: Frailty Status, Not Just Age, is Associated With Postoperative Opioid Consumption: A Retrospective, Population-based Analysis
Source: Ann Surg Open. 2024 Oct 4;5(4):e496. doi: 10.1097/AS9.0000000000000496 (PMC11661759; doi:10.1097/AS9.0000000000000496)
Supplement: Supplementary file 1 [file as9-5-e496-s001.pdf]

Supplement Table 1: Characteristics of the study cohort

			Score from mFi-5 criteria			P value
		Total	0	1	>=2	
		N=34,854	N=20,623	N=10,596	N=3,635	
Age (in years), mean (SD)		52.9 (16.15)	47.0 (15.35)	60.8 (13.53)	63.6 (11.72)	<0.001
Age >=65		9,306 (26.7%)	2,994 (14.5%)	4,477 (42.3%)	1,835 (50.5%)	<0.001
Gender N(%)	Male	15,421 (44.2%)	8,303 (40.3%)	5,212 (49.2%)	1,906 (52.4%)	<0.001
	Female	19,433 (55.8%)	12,320 (59.7%)	5,384 (50.8%)	1,729 (47.6%)	
Race N(%)	White, non-Hispanic	28,183 (80.9%)	16,838 (81.6%)	8,548 (80.7%)	2,797 (76.9%)	
	Black, non-Hispanic	3,136 (9.0%)	1,568 (7.6%)	1,097 (10.4%)	471 (13.0%)	
	Hispanic	898 (2.6%)	615 (3.0%)	198 (1.9%)	85 (2.3%)	
	Other	385 (1.1%)	252 (1.2%)	91 (0.9%)	42 (1.2%)	
	Unknown	2,252 (6.5%)	1,350 (6.5%)	662 (6.2%)	240 (6.6%)	
Insurance N(%)	Private	19,101 (54.8%)	13,297 (64.5%)	4,620 (43.6%)	1,184 (32.6%)	
	Medicare	8,967 (25.7%)	2,933 (14.2%)	4,298 (40.6%)	1,736 (47.8%)	
	Medicaid	5,277 (15.1%)	3,539 (17.2%)	1,248 (11.8%)	490 (13.5%)	
	Medicare and Medicaid	535 (1.5%)	182 (0.9%)	211 (2.0%)	142 (3.9%)	
	Uninsured/Other	974 (2.8%)	672 (3.3%)	219 (2.1%)	83 (2.3%)	
ASA class N(%)	1	3,261 (9.4%)	3,217 (15.6%)	41 (0.4%)	3 (0.1%)	
	2	20,217 (58.0%)	13,881 (67.3%)	5,484 (51.8%)	852 (23.4%)	
	3	10,843 (31.1%)	3,424 (16.6%)	4,833 (45.6%)	2,586 (71.1%)	
	4 or 5	520 (1.5%)	94 (0.5%)	234 (2.2%)	192 (5.3%)	
	Unknown	13 (0.0%)	7 (0.0%)	4 (0.0%)	2 (0.1%)	
BMI N(%)	<18.5	317 (0.9%)	207 (1.0%)	83 (0.8%)	27 (0.7%)	
	18.5 to 24.9	7,229 (20.7%)	5,154 (25.0%)	1,672 (15.8%)	403 (11.1%)	
	25 to 29.9	11,221 (32.2%)	6,844 (33.2%)	3,391 (32.0%)	986 (27.1%)	
	>=30	16,019 (46.0%)	8,373 (40.6%)	5,436 (51.3%)	2,210 (60.8%)	
	Unknown	68 (0.2%)	45 (0.2%)	14 (0.1%)	9 (0.2%)	
Cancer N(%)		2,046 (5.9%)	910 (4.4%)	786 (7.4%)	350 (9.6%)	<0.001

Tobacco use N(%)		6,413 (18.4%)	3,955 (19.2%)	1,745 (16.5%)	713 (19.6%)	<0.001
Inpatient N(%)		16,279 (46.7%)	9,314 (45.2%)	5,023 (47.4%)	1,942 (53.4%)	<0.001
Surgical priority N(%)	Elective	26,575 (76.2%)	15,278 (74.1%)	8,428 (79.5%)	2,869 (78.9%)	
	Emergent/Urgent	8,279 (23.8%)	5,345 (25.9%)	2,168 (20.5%)	766 (21.1%)	
Procedure type N(%)	Carotid Endarterectomy	89 (0.3%)	7 (0.0%)	47 (0.4%)	35 (1.0%)	
	Creation, Re-siting, or Closure of Ileostomy or Colostomy	89 (0.3%)	34 (0.2%)	38 (0.4%)	17 (0.5%)	
	Laparoscopic Anti-Reflux and Hiatal Hernia Surgery	545 (1.6%)	259 (1.3%)	223 (2.1%)	63 (1.7%)	
	Laparoscopic Appendectomy	3,606 (10.3%)	2,690 (13.0%)	735 (6.9%)	181 (5.0%)	
	Laparoscopic Cholecystectomy	9,312 (26.7%)	5,660 (27.4%)	2,651 (25.0%)	1,001 (27.5%)	
	Laparoscopic Colectomy	1,262 (3.6%)	585 (2.8%)	461 (4.4%)	216 (5.9%)	
	Minor Hernia	10,314 (29.6%)	5,755 (27.9%)	3,505 (33.1%)	1,054 (29.0%)	
	Major Hernia	1,364 (3.9%)	632 (3.1%)	505 (4.8%)	227 (6.2%)	
	Open Appendectomy	206 (0.6%)	142 (0.7%)	49 (0.5%)	15 (0.4%)	
	Open Cholecystectomy	199 (0.6%)	77 (0.4%)	72 (0.7%)	50 (1.4%)	
	Open Colectomy	697 (2.0%)	334 (1.6%)	256 (2.4%)	107 (2.9%)	
	Open Small Bowel Resection or Enterolysis	284 (0.8%)	157 (0.8%)	101 (1.0%)	26 (0.7%)	
	Thyroidectomy	728 (2.1%)	411 (2.0%)	234 (2.2%)	83 (2.3%)	
	Abdominal Hysterectomy	1,060 (3.0%)	658 (3.2%)	303 (2.9%)	99 (2.7%)	
	Laparoscopic Hysterectomy	3,027 (8.7%)	1,939 (9.4%)	846 (8.0%)	242 (6.7%)	
	Vaginal Hysterectomy	1,437 (4.1%)	1,010 (4.9%)	342 (3.2%)	85 (2.3%)	
	Other	635 (1.8%)	273 (1.3%)	228 (2.2%)	134 (3.7%)	
Postop complications N(%)		978 (2.8%)	495 (2.4%)	332 (3.1%)	151 (4.2%)	<0.001
Readmission N(%)		992 (2.8%)	482 (2.3%)	337 (3.2%)	173 (4.8%)	<0.001
Opioid related readmissions N(%)		55 (0.2%)	29 (0.1%)	19 (0.2%)	7 (0.2)	0.614
ED visit N(%)		2,316 (6.6%)	1,345 (6.5%)	702 (6.6%)	269 (7.4%)	0.146
Opioid related ED visit N(%)		410 (1.2%)	243 (1.2%)	123 (1.2%)	44 (1.2%)	0.971
Reoperation N(%)		532 (1.5%)	258 (1.3%)	195 (1.8%)	79 (2.2%)	<0.001
