# Supplementary material for: Frailty Status, Not Just Age, is Associated With Postoperative Opioid Consumption: A Retrospective, Population-based Analysis
Source: Ann Surg Open. 2024 Oct 4;5(4):e496. doi: 10.1097/AS9.0000000000000496 (PMC11661759; doi:10.1097/AS9.0000000000000496)
Supplement: Supplementary file 2 [file as9-5-e496-s002.pdf]

Supplement Table 2: Sensitivity analysis after excluding patients with complications, readmissions, emergency department visits and/or reoperations. Table represents results from a linear regression model for total opioid consumption in oral morphine equivalents.

	**Coefficient**	***P* value**	**95% CI**	
Frailty, mFi criteria score (ref group: 0)				
1	0.425	0.458	-0.699	1.549
>=2	3.316	0.000	1.489	5.144
Age (ref group: <45)				
45-64	-7.349	0.000	-8.501	-6.198
>=65	-19.734	0.000	-21.835	-17.634
Gender (ref group: Female)				
Male	-1.039	0.063	-2.134	0.056
Race (ref group: White, non-hispanic)				
Black,non-hispanic	11.364	0.000	9.428	13.300
Hispanic	4.327	0.002	1.523	7.131
Other	-0.253	0.904	-4.342	3.836
Unknown	0.338	0.695	-1.350	2.026
Insurance (ref group: Private)				
Medicare	2.888	0.002	1.052	4.724
Medicaid	8.465	0.000	6.864	10.066
Medicare and Medicaid	19.946	0.000	13.368	26.525
Uninsured/Other	3.887	0.008	1.001	6.773
ASA class (ref group: ASA class 2)				
ASA class 1	-2.898	0.000	-4.298	-1.499
ASA class 3	1.946	0.001	0.816	3.077
ASA class 4 or 5	5.598	0.018	0.969	10.228
Unknown	-6.659	0.646	-35.089	21.771
BMI (ref group: 18.5 to 24.9)				
<18.5	0.570	0.798	-3.800	4.939
25 to 29.9	0.749	0.213	-0.430	1.928
>=30	2.452	0.000	1.188	3.715
Unknown	-2.577	0.524	-10.503	5.349
Cancer	-3.803	0.003	-6.313	-1.292
Tobacco use	10.634	0.000	9.144	12.125
Inpatient	3.286	0.001	1.317	5.254
Surgical priority (ref group: Elective)				
Emergent/Urgent	-1.689	0.171	-4.109	0.731
Procedure type (ref group: Laparoscopic Cholecystectomy)				
Carotid Endarterectomy	11.015	0.240	-7.350	29.379
Creation, Re-siting, or Closure of Ileostomy or Colostomy	16.078	0.022	2.345	29.812
Laparoscopic Anti-Reflux and Hiatal Hernia Surgery	-9.745	0.002	-15.781	-3.710
Laparoscopic Appendectomy	0.082	0.917	-1.458	1.622
Laparoscopic Colectomy	1.503	0.389	-1.917	4.922
Minor Hernia	6.631	0.000	5.193	8.068
Major Hernia	8.339	0.000	5.503	11.175
Open Appendectomy	2.763	0.438	-4.214	9.740
Open Cholecystectomy	20.526	0.002	7.439	33.613

Open Colectomy	7.010	0.011	1.610	12.409
Open Small Bowel Resection or Enterolysis	3.232	0.338	-3.375	9.840
Thyroidectomy	-9.293	0.000	-12.300	-6.286
Abdominal Hysterectomy	6.099	0.018	1.065	11.134
Laparoscopic Hysterectomy	-0.745	0.621	-3.700	2.210
Vaginal Hysterectomy	-0.304	0.866	-3.837	3.230
Other	8.044	0.005	2.437	13.651
Prescribed OME	0.344	0.000	0.241	0.446
