# Supplementary material for: Frailty Status, Not Just Age, is Associated With Postoperative Opioid Consumption: A Retrospective, Population-based Analysis
Source: Ann Surg Open. 2024 Oct 4;5(4):e496. doi: 10.1097/AS9.0000000000000496 (PMC11661759; doi:10.1097/AS9.0000000000000496)
Supplement: Supplementary file 3 [file as9-5-e496-s003.pdf]

Supplement Table 3: Sensitivity analysis after excluding patients with complications, readmissions, emergency department visits and/or reoperations. Table represents results from a linear regression model for total opioid consumption in oral morphine equivalents for patients all 65 years old and older.

	**Coefficient**	***P* value**	**95% CI**	
Frailty, mFi criteria score (ref group: 0)				
1	-1.179	0.159	-2.821	0.463
>=2	2.725	0.032	0.241	5.209
Gender (ref group: Female)				
Male	-2.189	0.026	-4.114	-0.263
Race (ref group: White, non-hispanic)				
Black,non-hispanic	14.031	0.000	9.702	18.361
Hispanic	3.333	0.355	-3.738	10.404
Other	-2.228	0.539	-9.334	4.879
Unknown	0.166	0.931	-3.606	3.938
Insurance (ref group: Private)				
Medicare	-1.397	0.237	-3.715	0.920
Medicaid	12.575	0.067	-0.872	26.022
Medicare and Medicaid	16.696	0.000	9.158	24.234
Uninsured/Other	-4.110	0.142	-9.590	1.371
ASA class (ref group: ASA class 2)				
ASA class 1	-2.518	0.218	-6.526	1.489
ASA class 3	1.793	0.027	0.203	3.383
ASA class 4 or 5	7.594	0.014	1.560	13.629
Unknown	29.704	0.160	-11.764	71.171
BMI (ref group: 18.5 to 24.9)				
<18.5	5.110	0.269	-3.960	14.179
25 to 29.9	0.091	0.924	-1.772	1.954
>=30	3.608	0.001	1.469	5.747
Unknown	2.710	0.787	-16.941	22.361
Cancer	-2.433	0.140	-5.665	0.800
Tobacco use	11.886	0.000	8.160	15.611
Inpatient	3.187	0.031	0.297	6.077
Surgical priority (ref group: Elective)				
Emergent/Urgent	-2.714	0.137	-6.294	0.866
Procedure type (ref group: Laparoscopic Cholecystectomy)				
Carotid Endarterectomy	11.072	0.388	-14.094	36.238
Creation, Re-siting, or Closure of Ileostomy or Colostomy	10.045	0.150	-3.617	23.707
Laparoscopic Anti-Reflux and Hiatal Hernia Surgery	-6.418	0.026	-12.073	-0.763
Laparoscopic Appendectomy	-0.640	0.678	-3.656	2.377
Laparoscopic Colectomy	2.330	0.317	-2.231	6.891
Minor Hernia	5.889	0.000	3.723	8.054
Major Hernia	7.276	0.001	3.116	11.435
Open Appendectomy	9.628	0.252	-6.847	26.102
Open Cholecystectomy	13.254	0.016	2.441	24.066
Open Colectomy	7.395	0.032	0.626	14.164

Open Small Bowel Resection or Enterolysis	7.789	0.126	-2.196	17.774
Thyroidectomy	-6.008	0.079	-12.720	0.705
Abdominal Hysterectomy	1.356	0.758	-7.282	9.994
Laparoscopic Hysterectomy	-1.480	0.516	-5.947	2.987
Vaginal Hysterectomy	-4.450	0.078	-9.396	0.496
Other	10.809	0.011	2.451	19.168
Prescribed OME	0.130	0.005	0.038	0.221
